# Phenotype–genotype correlation in patients with typical and atypical branchio-oto-renal syndrome

**DOI:** 10.1038/s41598-022-04885-w

**Published:** 2022-01-19

**Authors:** Masatsugu Masuda, Ayako Kanno, Kiyomitsu Nara, Hideki Mutai, Naoya Morisada, Kazumoto Iijima, Noriko Morimoto, Atsuko Nakano, Tomoko Sugiuchi, Yasuhide Okamoto, Sawako Masuda, Sayaka Katsunuma, Kaoru Ogawa, Tatsuo Matsunaga

**Affiliations:** 1 Department of Otolaryngology, Kyorin University, Tokyo, Japan; 2 Division of Hearing and Balance Research, National Institute of Sensory Organs, National Hospital Organization Tokyo Medical Center, 2-5-1 Higashigaoka Meguro-ku, Tokyo, 152-8902 Japan; 3 Department of Otolaryngology, Nippon Koukan Hospital, Kanagawa, Japan; 4 Department of Clinical Genetics, Hyogo Prefectural Kobe Children’s Hospital, Hyogo, Japan; 5 Department of Pediatrics, Kobe University Graduate School of Medicine, Hyogo, Japan; 6 Hyogo Prefectural Kobe Children’s Hospital, Hyogo, Japan; 7 Department of Advanced Pediatric Medicine, Kobe University Graduate School of Medicine, Hyogo, Japan; 8 Department of Otolaryngology, National Center for Child Health and Development, Tokyo, Japan; 9 Division of Otolaryngology, Chiba Children’s Hospital, Chiba, Japan; 10 Department of Otolaryngology, Kanto Rosai Hospital, Kanagawa, Japan; 11 Department of Otolaryngology, Tokyo Saiseikai Central Hospital, Tokyo, Japan; 12 Department of Otolaryngology, National Hospital Organization Mie National Hospital, Mie, Japan; 13 Department of Otolaryngology, Hyogo Prefectural Kobe Children’s Hospital, Hyogo, Japan; 14 Department of Otolaryngology, Keio University School of Medicine, Tokyo, Japan; 15 Medical Genetics Center, National Hospital Organization Tokyo Medical Center, Tokyo, Japan

**Keywords:** Disease genetics, Genetics research, Disease genetics, Medical genetics, Inner ear, Paediatric research

## Abstract

Some patients have an atypical form of branchio-oto-renal (BOR) syndrome, which does not satisfy the diagnostic criteria, despite carrying a pathogenic variant (P variant) or a likely pathogenic variant (LP variant) of a causative gene. P/LP variants phenotypic indices have yet to be determined in patients with typical and atypical BOR syndrome. We hypothesized that determining phenotypic and genetic differences between patients with typical and atypical BOR syndrome could inform such indices. Subjects were selected from among patients who underwent genetic testing to identify the cause of hearing loss. Patients were considered atypical when they had two major BOR diagnostic criteria, or two major criteria and one minor criterion; 22 typical and 16 atypical patients from 35 families were included. Genetic analysis of *EYA1*, *SIX1*, and *SIX5* was conducted by direct sequencing and multiplex ligation-dependent probe amplification. *EYA1* P/LP variants were detected in 25% and 86% of atypical and typical patients, respectively. Four *EYA1* P/LP variants were novel. Branchial anomaly, inner ear anomaly, and mixed hearing loss were correlated with P/LP variants. Development of refined diagnostic criteria and phenotypic indices for atypical BOR syndrome will assist in effective detection of patients with P/LP variants among those with suspected BOR syndrome.

## Introduction

Branchio-oto-renal (BOR) syndrome is an autosomal dominant disorder characterized by branchial anomalies (branchial cleft or sinus, preauricular pits, or auricular deformity), hearing loss, and renal anomalies. The prevalence of BOR syndrome in European populations is estimated at one case per 40,000, and the syndrome is responsible for 2% of profound deafness in children^1^. BOR syndrome has a high penetrance, with variable expressivity^2^, and the known causative genes are *EYA1* (8q13.3), *SIX1* (14q23.1), and *SIX5* (19q13.32)^3–5^. The mutation detection rate in patients with BOR syndrome is 40–75% for *EYA1*^6–9^, 2% for *SIX1*^7,8^, and 0–3.1% for *SIX5*^4,7,8^. *EYA1* comprises 16 coding exons and encodes a 559 amino acid protein^5^, which is expressed between the fourth and sixth weeks of human development, and is involved in the formation of the kidney and first and second branchial arches^5,10^. *SIX1* interacts with *EYA1* and participates in the development of the eye, inner ear, and kidney^11^, while SIX5 protein binds to EYA1 protein and activates gene transcription^4^.

Hearing loss is the most common symptom of BOR syndrome, with 64–100% of patients having some degree of hearing loss^6,7,9,12,13^. Mixed hearing loss is the most common type of hearing loss in patients with BOR syndrome (40–52%), followed by sensorineural (25–30%) and conductive (19–33%) hearing loss^2,9,13,14^, and imaging studies can detect anomalies of the inner ear (18–92%) and/or middle ear (15–100%)^7,15^. Cochlear hypoplasia is the most common inner ear anomaly (33–100%), followed by enlarged vestibular aqueduct (EVA) (24–50%) and internal auditory canal anomalies (17–86%)^2,16,17^. Other than hearing loss, preauricular pits (53–83%), renal anomalies (38–70%), branchial anomalies (49–73%), and auricular deformities (32–36%) are included among the major diagnostic criteria for BOR syndrome^2,6,7,9,12,13^. Of these, renal anomalies are a substantial cause of morbidity during the lifespan of patients with the condition^18^.

According to the diagnostic criteria defined by Chang et al*.* and Smith (original diagnostic criteria)^6,8^, patients are diagnosed with typical BOR syndrome when they meet three major criteria (hearing loss, preauricular pits, branchial anomalies, renal anomalies, or auricular deformities), two major criteria with two minor criteria (inner ear anomalies, middle ear anomalies, external auditory canal anomalies, preauricular tags, facial asymmetry, or palatal anomalies), or one major criterion with an affected first-degree relative meeting the above criteria for typical BOR syndrome. There are some reports of patients with atypical BOR syndrome, which does not satisfy the clinical criteria for typical BOR syndrome, despite carrying *EYA1* or *SIX1* mutations^12,15,19^. In this report, we refer to patients with typical or atypical BOR syndrome as typical or atypical patients, respectively.

The identification of a pathogenic variant (P variant) or a likely pathogenic variant (LP variants) in a causative gene is important for accurate genetic counseling. Further, genetic screening limited to typical patients alone will miss P/LP variants in atypical patients; however, conducting genetic analyses for all atypical patients is unlikely to be cost-effective. We hypothesized that determining phenotypic and genetic differences between typical and atypical patients may identify some indices related to P/LP variants. For this purpose, we analyzed the phenotypic and genetic features of patients with typical and atypical BOR syndrome.

## Results

### Overview of genetic analysis

**Figure 1:**
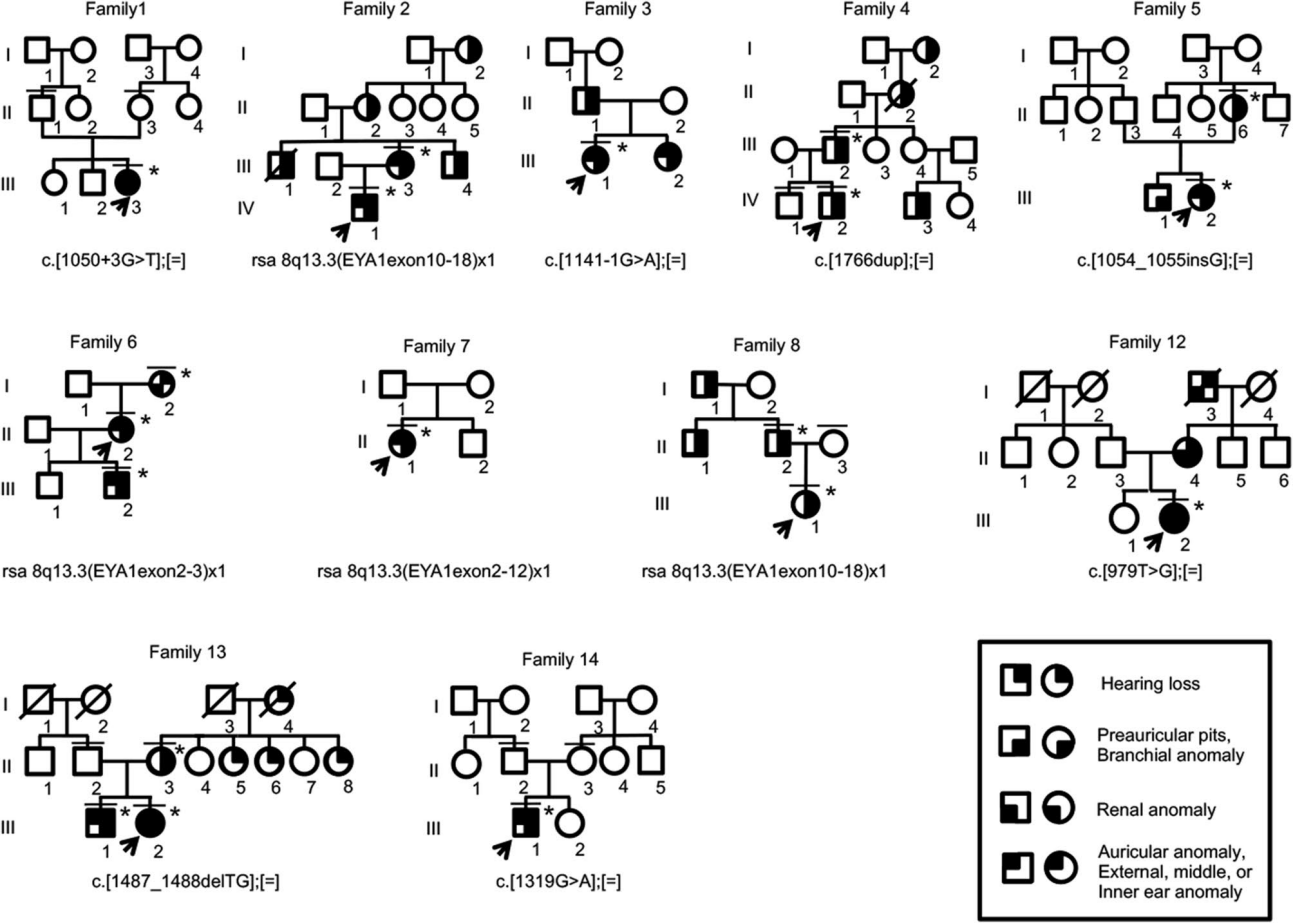
The pedigree of families with typical BOR syndrome and with P/LP variants. Arrows, probands; asterisks, patients with P/LP variants; crossbars, patients who underwent genetic analysis.

**Figure 2:**
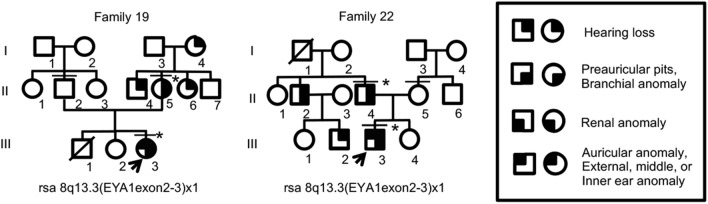
The pedigree of families with atypical BOR syndrome and with P/LP variants. For the meaning of the symbols, see the legend of Fig. 1.

Patients were diagnosed with typical BOR syndrome when they met the original diagnostic criteria^6,8^. In addition to typical patients, we diagnosed patients with atypical BOR when they had two major criteria, or two major criteria and one minor criterion. No families contained both typical and atypical patients. Genetic analysis was conducted for 22 typical patients from 14 families, 16 atypical patients from 12 families, and 18 asymptomatic family members (crossbars in Figs. 1 and 2, and Supplementary Figs. S1 and S2). *GJB2* and mitochondrial m.1555A>G and m.3243A>G variants, which are most frequently associated with hearing loss in Japanese populations^20,21^, were not detected. *EYA1* P/LP variants were identified in 23/38 (61%) typical and atypical patients (Tables 1 and 2, asterisks in Figs. 1 and 2). P/LP variants consisted of two missense variants (15%), two splice site variants (15%), three frameshift deletions (23%), and six large deletions (46%) (Tables 1 and 2, Supplementary Fig. S3). Two novel P variants (c.1054_1055insG, and c.1487_1488delTG) and two novel LP variants (c.1050+3G>T, c.979T>G) were detected. No *SIX1* or *SIX5* P/LP variants were identified. Among three sporadic cases with LP variants (Families 1, 7, and 14), parent samples were available for two (Families 1 and 14), in which LP variants were determined to be de novo. The large deletions detected by multiplex ligation-dependent probe amplification (MLPA)^12^ were classified into 3 types of copy number variants (CNVs); rsa 8q13.3(EYA1exon10-18) × 1 (c.(743_966+9)_(*76_?)del), rsa 8q13.3(EYA1exon2-3) × 1 (c.(?_-6)_(61_187)del), and rsa 8q13.3(EYA1exon2-12) × 1 (c.(?_-6)_(1114_1199 + 34)del). All the variants were curated and described in Supplementary Table S1.

**Table 1:** Phenotypes and variants in patients with typical BOR syndrome.

Family no.	Patient no.	Sex	FM with typical BOR	FM with atypical BOR	HL	BA	PP	RA	AD	Other clinical findings	Nucleotide change^$^	Protein change^$^	Pathogenicity	Variant inheritance	Reference
1	III-3	F	−	−	+	+	−	+	+	NT	c.1050+3G>T	(Splice site variant)	Likely pathogenic	De novo	This study
2	IV-1	M	+	−	+	+	+	−	−	NT	rsa 8q13.3(EYA1exon10-18) × 1^&^	Unknown	Likely pathogenic	I	Unzaki et al.^7^
	III-3	F			+	+	+	−	−	IEA^✝^, MEA				U	
3	III-1	F	+	−	+	+	+	−	−	IEA^✝^, MEA	c.1141-1G>A	(Splice site variant)	Pathogenic	U	Sanggaard et al.^29^
4	IV-2	M	+	−	+	+	+	−	−	NT	c.1766dup	p.Glu590Glyfs*42	Likely pathogenic	I	Matsunaga et al.^30^
	III-2	M			+	−	+	−	−	NT				U	
5	III-2	F	+	−	+	+	+	−	−	IEA*^✝^	c.1054_1055insG	p.Pro352Argfs*26	Pathogenic	I	This study
	II-6	F			+	+	+	−	−	NT				U	
6	II-2	F	+	−	+	−	+	−	+	FA, MEA, IEA^✝^	rsa 8q13.3(EYA1exon2-3) × 1	Unknown	Likely Pathogenic	I	Unzaki et al.^7^
	III-2	M			+	−	+	−	+	EEA				I	
	I-2	F			+	−	−	+	−	NT				U	
7	II-1	F	−	−	+	+	+	−	−	PT, IEA^✝^	rsa 8q13.3(EYA1exon2-12) × 1	Unknown	Pathogenic	U	Unzaki et al.^7^
8	III-1	F	+	−	+	+	+	−	−	-	rsa 8q13.3(EYA1exon10-18) × 1	Unknown	Likely pathogenic	I	Unzaki et al.^7^
	II-2	M			+	+	+	−	−	NT				U	
9	III-3	M	−	−	+	+	−	−	+	MEA	None detected	None detected	–	–	–
10	III-1	F	−	−	+	−	+	+	−	EEA, IEA^✝^, MEA	None detected	None detected	–	–	–
11	II-1	F	−	−	+	−	+	+	−	IEA*^✝^	None detected	None detected	–	–	–
12	III-2	F	+	−	+	+	+	+	−	IEA*^✝^, MEA	c.979T>G	p.Trp327Gly	Likely pathogenic	U	This study
13	III-2	F	+	−	+	+	+	+	+	IEA*^✝^, MEA	c.1487_1488delTG	p.Val496Glufs*35	Pathogenic	I	This study
	III-1	M			+	+	+	−	+	IEA^✝^, MEA				I	
	II-3	F			+	+	+	−	−	NT				U	
14	III-1	M	−	−	+	+	−	−	−	IEA^✝^, MEA	c.1319G > A	p.Arg440Gln	Likely pathogenic	De novo	Kumar et al*.*^31^

*F* female, *M* male, *FM* family member, *HL* hearing loss, *BA* branchial anomalies, *PP* preauricular pits, *RA* renal anomaly, *AD* auricular deformity, *EEA* external ear anomaly, *FA* facial asymmetry, *IEA* inner ear anomaly, *MEA* middle ear anomaly, *NT* not tested, *PT* preauricular tag, *I* inherited, *U* unknown. *Includes enlarged vestibular aqueduct. ^✝^Includes cochlear hypoplasia. ^$^Reference sequences for nucleotide numbering and protein numbering are NM_000503.5 and NP_000494.2, respectively. ^&^The results of MLPA are presented using the International System for Human Cytogenomic Nomenclature (2016)^29^.

**Table 2:** Phenotypes and variants in patients with atypical BOR syndrome.

Family no	Patient no	Sex	FM with typical BOR	FM with atypical BOR	HL	BA	PP	RA	AD	Other clinical findings	Nucleotide change^$^	Protein change^$^	Pathogenicity	Variant inheritance	Reference
15	II-2	F	−	−	+	−	+	−	−	IEA^✝^	None detected	None detected	–	–	–
16	II-1	F	−	+	+	−	+	−	−	–	None detected	None detected	–	–	–
	II-2	M			+	−	+	−	−	–	None detected	None detected	–	–	–
17	III-1	M	−	−	+	−	+	−	−	NT	None detected	None detected	–	–	–
18	II-1	F	−	−	+	−	+	−	−	–	None detected	None detected	–	–	–
19	III-3	F	−	+	+	−	+	−	−	IEA*^✝^	rsa 8q13.3(EYA1exon2-3) × 1	Unknown	Likely pathogenic	I	Unzaki et al.^7^
	II-5	F			+	−	+	−	−	NT				U	
20	III-1	M	−	−	+	−	−	−	+	MEA	None detected	None detected	–	–	–
21	II-1	F	−	−	+	−	−	+	−	–	None detected	None detected	–	–	–
22	III-3	M	−	+	+	−	+	−	−	IEA*^✝^	rsa 8q13.3(EYA1exon2-3) × 1	Unknown	Likely pathogenic	I	Unzaki et al.^7^
	II-4	M			+	+	−	−	−	NT				U	
23	III-1	M	−	−	+	−	−	+	−	IEA	None detected	None detected	–	–	–
24	III-1	F	−	−	+	−	+	−	−	NT	None detected	None detected	–	–	–
25	III-1	M	−	−	+	−	+	−	−	NT	None detected	None detected	–	–	–
26	III-1	F	−	+	+	−	+	−	−	NT	None detected	None detected	–	–	–
	II-2	F			+	−	+	−	−	NT	None detected	None detected	–	–	–

*F* female, *M* male, *FM* family member, *HL* hearing loss, *BA* branchial anomalies, *PP* preauricular pits, *RA* renal anomaly, *AD* auricular deformity, *EEA* external ear anomaly, *FA* facial asymmetry, *IEA* inner ear anomaly, *MEA* middle ear anomaly, *NT* not tested, *PT* preauricular tag, *I* inherited, *U* unknown. *Includes enlarged vestibular aqueduct. ^✝^Includes cochlear hypoplasia. ^$^Reference sequences for nucleotide numbering and protein numbering are NM_000503.5 and NP_000494.2, respectively.

### Differences in phenotypes with the same variant

The same copy number variant, rsa 8q13.3(EYA1exon2-3) × 1 was detected in patients with the typical (Family 6) and atypical (Family 19 and 22) BOR syndrome. Furthermore, phenotypes were different even within the family. Other families with c.1766dup (Family 4) and c.1487_1488delTG (Family 13) also showed differences in phenotypes within the family.

### Difference in the rate of P/LP variants detection between typical and atypical patients

**Figure 3:**
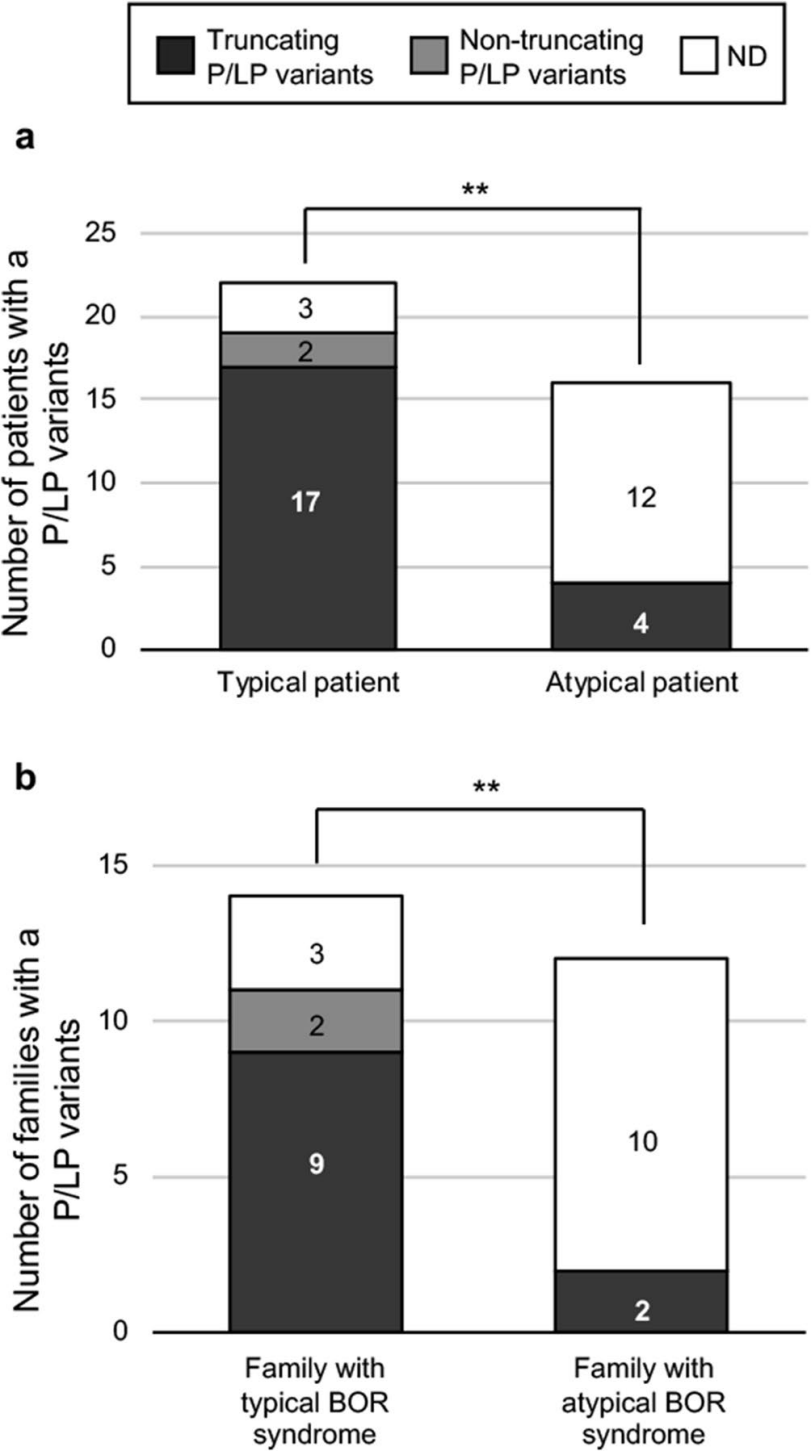
Numbers of patients/families with P/LP variants. (**a**) Patients with typical and atypical BOR syndrome. (**b**) Families with typical and atypical BOR syndrome. **Significant difference at p < 0.01 (Fisher’s exact test). ND, P/LP variants were not detected.

There was a higher P/LP variants detection rate in typical patients (86%), who met the diagnostic criteria for BOR syndrome, than in atypical patients (33%), who did not fully meet the diagnostic criteria (p = 0.0001) (Fig. 3a). Truncating or non-truncating P/LP variants were not associated with typical or atypical BOR syndrome. Reflecting the high prevalence of P/LP variants in typical patients, the detection rate of P/LP variants was significantly higher in families with typical BOR syndrome (79%) than in those with atypical BOR syndrome (17%) (p = 0.0016) (Fig. 3b). The number of families with non-truncating P/LP variants was less than that with truncating variants, accounting for 15% of all families with P/LP variants.

### Differences in phenotypes between patients with typical and atypical BOR syndrome

**Figure 4:**
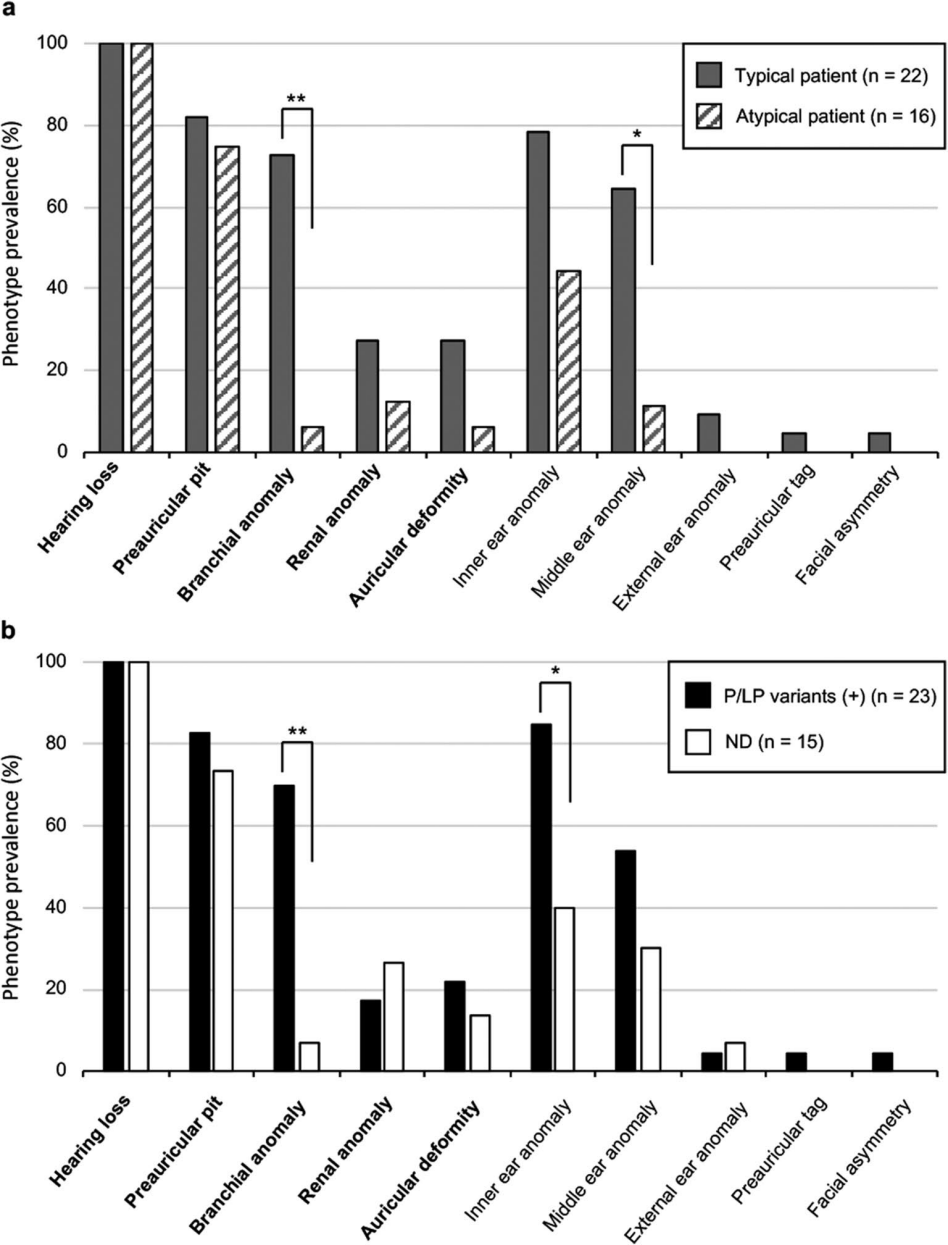
Prevalence rates of phenotypes in patients. (**a**) Patients with typical or atypical BOR syndrome. (**b**) Patients with or without P/LP variants of *EYA1*. Significant difference at **p < 0.01 and *p < 0.05, respectively (Fisher’s exact test).

As the subjects in the present study were selected from patients who underwent genetic testing to identify the cause of hearing loss, all patients had hearing loss (Fig. 4a). Among major diagnostic criteria (Fig. 4a, written in bold letters), branchial anomalies were significantly more common in typical (72%) than atypical (6%) patients (p = 0.0001). Preauricular pits were frequently identified in both typical and atypical patients, with no significant difference between the groups (p = 0.6984). In addition, there were no significant differences in the prevalence of renal and auricular anomalies between typical and atypical patients (p = 0.4262 and 0.2029, respectively).

Temporal bone anomalies, which are among the minor diagnostic criteria, were evaluated by temporal bone CT imaging in 14 typical and nine atypical patients. Middle ear anomalies were more prevalent in typical (64%) than in atypical (11%) patients (p = 0.0273) (Fig. 4a). The prevalence of inner ear anomalies was the third most prevalent symptom in both typical and atypical patients, and there was no significant difference between the groups (p = 0.1816). Overall, the only significant phenotypic differences between patients with typical and atypical BOR syndrome were branchial and middle ear anomalies.

### Differences in phenotypes between patients with and without P/LP variants

To identify characteristic phenotypes in patients with P/LP variants, we compared the prevalence of each symptom in patients with and without P/LP variants (Fig. 4b). Among major criteria, brachial anomalies were more prevalent in patients with P/LP variants (70%) than in those without P/LP variants (6.7%) (p = 0.0002). The prevalence rates of renal and auricular anomalies were not high (17% and 22%, respectively), even among patients with P/LP variants. Among minor criteria, inner ear anomalies were more prevalent in patients with P/LP variants (85%) than in those without P/LP variants (40%) (p = 0.0393); however, there was no significant difference in the prevalence of the middle ear anomalies between patients with (54%) and without P/LP variants (30%) (p = 0.3673).

**Figure 5:**
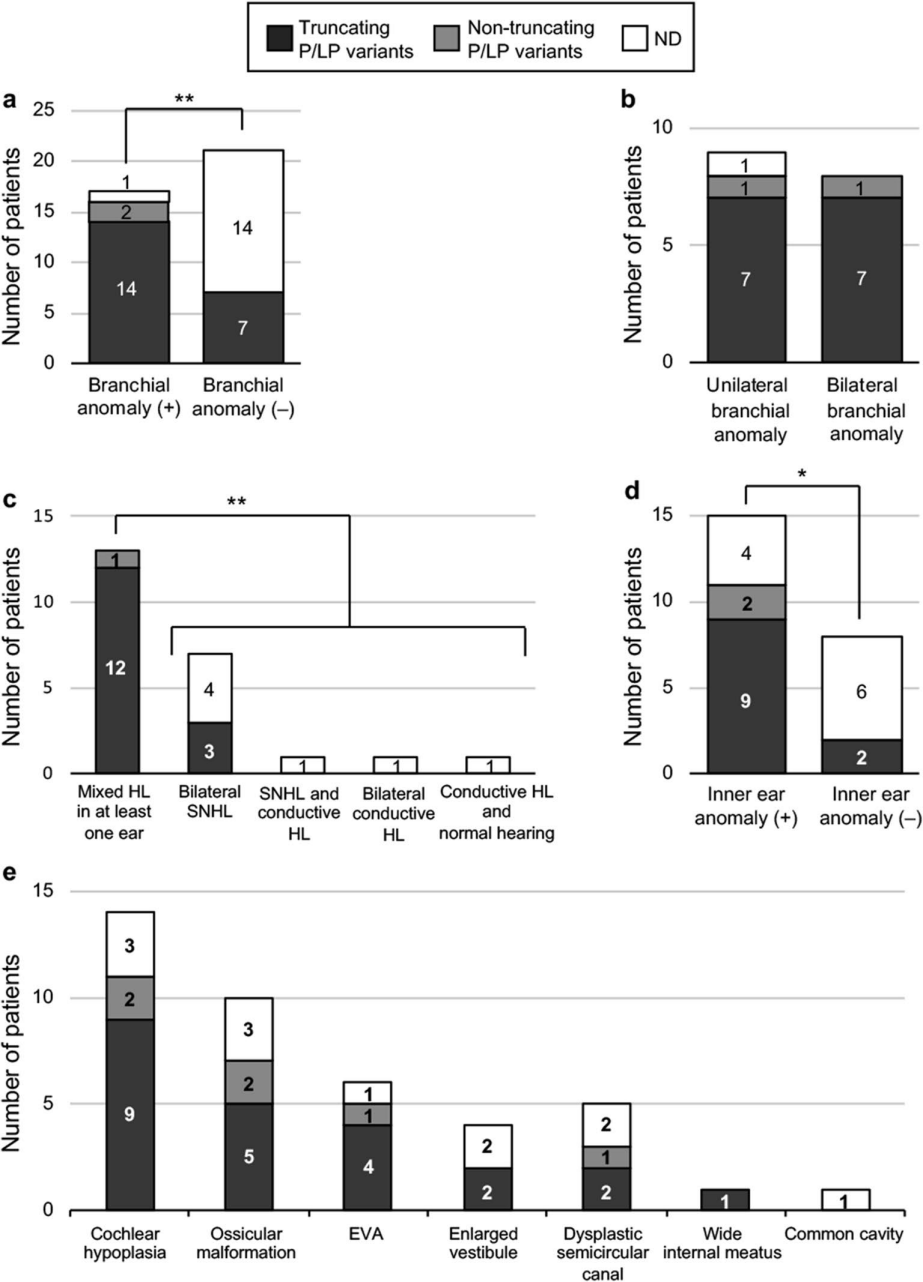
Relationship of P/LP variants with phenotypes. (**a**) Association of branchial anomaly with P/LP variants. (**b**) Relationship between P/LP variants and unilateral or bilateral branchial anomaly. (**c**) Association of hearing loss types with P/LP variants. (**d**) Association of inner ear anomalies with P/LP variants. (**e**) Association of ear anomalies with P/LP variants. Significant difference at **p < 0.01 and *p < 0.05, respectively (Fisher’s exact test).

There was a higher detection rate of P/LP variants in patients with branchial anomalies (94%) than in those without such anomalies (33%) (p = 0.0002) (Fig. 5a). Whether the anomaly was bilateral or unilateral was not related to the type of P/LP variants (i.e., truncating or non-truncating) (Fig. 5b).

Mixed hearing loss in at least one ear was observed in 57% of patients, and bilateral sensorineural hearing loss (SNHL) was observed in 30% of patients. Other types of hearing loss observed were sensorineural and conductive in each ear (4%), bilateral conductive hearing loss (4%), and conductive hearing loss and normal hearing in each ear (4%) (Fig. 5c). P/LP variants were significantly more common in patients with mixed hearing loss (100%) than in those without mixed hearing loss (30%) (p = 0.0005). The severity of hearing loss was neither associated with PV/LPV nor with the type of P/LP variants (Supplementary Fig. S4).

P/LP variants were more frequently detected in patients with inner ear anomalies (85%) than in those without such anomalies (25%) (p = 0.0393) (Fig. 5d). By contrast, detection rates of P/LP variants did not differ significantly between patients with (7/10, 70%) and without middle ear anomalies (6/13, 46%) (p = 0.4015). Among inner ear anomalies, cochlear hypoplasia and EVA had the highest and second-highest prevalence rates (61% and 26%, respectively) in patients with typical or atypical BOR syndrome, and detection rates of P/LP variants were also high in patients with cochlear hypoplasia (78%) and EVA (84%) (Fig. 5e and Supplementary Table S2). Other inner ear anomalies accompanying P/LP variants were enlarged vestibule, dysplastic semicircular canal, and wide internal meatus; however, wide internal meatus may be just a normal variant of the internal meatus, as previously reported^22,23^.

Several atypical patients with phenotypes related to P/LP variants had *EYA1* P/LP variants. Family 19-III-3 and Family 22-III-3 had mixed hearing loss, cochlear hypoplasia, and EVA, and carried *EYA1* LP variants (Supplementary Fig. S5), despite being atypical. Another atypical patient (Family 22-II-4) with branchial anomaly also had LP variant.

### Prevalence of phenotypes in all patients and detection rates of P/LP variants in each phenotype

Inner ear anomaly, mixed hearing loss, and branchial anomaly had the third-, fourth-, and fifth-highest prevalence in all patients (65%, 57%, and 45%, respectively) following hearing loss (100%) and preauricular pit (79%) (Supplementary Fig. S6a). Moreover, the three phenotypes occupied the top three with a high detection rate of having P/LP variants (100%, 94%, and 73% for mixed hearing loss, branchial anomaly, and inner ear anomaly, respectively) except for preauricular tag and facial asymmetry, in which detection rates of variants in the two phenotypes were 100% (Supplementary Fig. S6b). However, there was only one patient each with each of the two phenotypes.

## Discussion

The phenotypic and genotypic characteristics of BOR syndrome, including those of atypical patients, have not been examined in detail. In the present study, we detected *EYA1* P/LP variants in 25% of patients with atypical BOR syndrome and in 86% of typical patients. Our results suggest that refining the diagnostic criteria for atypical BOR syndrome may assist in effective detection of patients with P/LP variants, even for atypical patients.

More P/LP variants were detected in atypical patients in this study than in past reports. For example, Orten et al*.* reported mutation detection rates of 31% and 7% in patients with typical and questionable BOR syndrome, respectively, using Sanger sequencing, among which all questionable patients had at least one of the major diagnostic criteria^19^. Krug et al*.* detected mutations in 76% and 9% of typical and atypical patients, who had at least two BOR syndrome features, respectively, by direct sequencing and MLPA^12^. The difference in the detection rate in typical patients can be explained by the use of MLPA, which can detect copy number variants that cannot be identified by the sequencing technologies used in the present study. Accordingly, 46% of *EYA1* P/LP variants were detected by MLPA in the present study.

The type of P/LP variants (i.e., truncating or non-truncating) was not associated with the phenotypic expression of BOR syndrome (i.e., typical or atypical), nor was it associated with phenotype severity, consistent with past reports^7,12,19^. The prevalence of renal anomalies in patients with P/LP variants was 17% in the present study, while the two previous studies reported rates of 53% and 70%^7,12^. By contrast, the prevalence of different P/LP variants types did not differ significantly among the three studies. These findings reinforce the notion that there are no phenotype–genotype correlations in patients with BOR syndrome.

Another finding is that the typical (Family 6) and atypical (Family 19 and Family 22) BOR syndrome patients carried the same copy number variant rsa 8q13.3 (EYA1exon2-3) × 1. Start codons of most of the transcriptional isoforms of *EYA1* are mapped on exon 2 or exon 3 except NM_001288575.2 (encoding isoform 5 (NP_001275504.1)) with its start codon at exon 6. The isoform 5 is N-terminal 117 amino acid residues shorter in its N-terminal region than that of NM_000503.5 (isoform 1C (NP_000494.2)). Neither functional property of isoform 5, nor whether the isoform is expressed in developing neck, ears or kidneys is known. The spectrum of BOR phenotypes in the patients with 8q13.3(EYA1exon2-3) × 1 could be explained, at least in part, by differential expression levels of intact *EYA1* from the other intact allele affected via non-coding variants in the promoter or enhancer, 5′ or 3′-untranslated, or intronic regions that influence gene expression*,* differential protein levels of isoform 5 or other hypothetical EYA1 protein isoforms from the CNV allele, or difference in activity of the EYA1 coactivators such as SIX1 protein. These possibilities are very speculative and would need extensive genetic analysis including non-coding regions. Another possibility of phenotypic variability observed even within a family might be due to maternal factors that modify phenotypes during embryonic and fetal development^12^.

Notably two de novo LP variants (c.1050 + 3G>T in Family 1-III-3 and c.1319G>A in Family 14-III-1) were detected. Previous studies also reported de novo mutations of *EYA1*^7,12^. These findings indicate that BOR syndrome should be considered in patients without a family history.

Our data demonstrate that branchial anomaly, inner ear anomaly, and mixed hearing loss are potential indicators of P/LP variants. As *EYA1* has roles in morphogenesis of the inner and middle ear structures^10^, it is logical to infer that P/LP variants in this gene will result in inner ear anomaly and mixed hearing loss, comprising simultaneous SNHL and conductive hearing loss; however, the prevalence of middle ear anomalies causing conductive hearing loss did not differ significantly between patients with and without P/LP variants. This may be due to the presence of subtle middle ear anomalies that cause conductive hearing loss but cannot be detected by CT imaging.

Branchial anomaly, inner ear anomaly, and mixed hearing loss were related to the high detection rate of P/LP variants in all patients, including both typical and atypical cases. Therefore, to detect patients with P/LP variants efficiently, even in patients with suspected BOR syndrome, the diagnostic criteria for suspected BOR syndrome, such as the criteria for atypical BOR syndrome that we defined, should first be made. In addition, it would be advisable to add the explanatory note that recommends genetic testing for patients with the three phenotypes listed above. One issue with the spectrum disorders like BOR syndrome is that some phenotypes can often be missed due to poor clinical evaluation. In particular, it is not easy to obtain a complete family history, but in BOR syndrome, the family history is critical for diagnosing typical cases. Therefore, typical patients might have been clinically misdiagnosed as atypical. Even in our patients, such cases cannot be completely excluded. By setting diagnostic criteria and the explanatory note for suspected BOR syndrome, genetic tests will be performed on clinically misdiagnosed patients, and in the end, more patients will be diagnosed correctly.

In summary, we report the detection rate of *EYA1* P/LP variants in patients with typical and atypical BOR syndrome. Further, we demonstrate that branchial anomaly, inner ear anomaly, and mixed hearing loss may be good indices of P/LP variants in patients with BOR syndrome or suspected BOR syndrome.

## Methods

### Subjects

Subjects were probands with typical or atypical BOR syndrome with hearing loss with the onset < 10 years old, and their family members. All probands underwent careful examinations of comorbidity in the department of pediatrics or nephrology in addition to otology. Other subjects also underwent physical and otologic examination as much as possible. All probands and/or their parents were interviewed to determine family history. This study was approved by the institutional ethics review board at the National Hospital Organization Tokyo Medical Center and the respective ethical committees of collaborating hospitals. Written informed consent was obtained from all subjects included in the study, or from their parents. All methods were performed in accordance with the Guidelines for Genetic Tests and Diagnosis in Medical Practice of the Japanese Association of Medical Sciences and the Declaration of Helsinki.

### Clinical evaluations

General medical reports were reviewed, and family histories were obtained by extended personal interviews of family members. Audiological examinations included pure tone audiometry, conditional orientation reflex audiometry, play audiometry, auditory brainstem responses, and auditory steady-state evoked responses. The severity and type of hearing loss were categorized based on recommendations by the GENDEAF study group^24^. Severity was based on the better hearing ear, and averaged over 0.5, 1, 2, and 4 kHz. Wave V response thresholds were used for evaluation of auditory brainstem response. Severity was categorized as follows: mild, 20–40 dB HL; moderate, 41–70 dB HL; severe, 71–95 dB HL; profound, > 95 dB HL. Types of hearing loss were defined as follows: (1) conductive, normal bone-conduction thresholds (< 20 dB), and an air–bone gap ≥ 15 dB HL averaged over 0.5, 1, and 2 kHz; (2) sensorineural, an air–bone gap < 15 dB HL averaged over the three frequencies; and (3) mixed, bone-conduction threshold > 20 dB HL, together with air–bone gap ≥ 15 dB HL, averaged over the three frequencies^24^. Temporal bone anomalies were assessed by temporal bone CT, when possible.

### Genetic analysis

Genomic DNA was extracted from peripheral blood samples using a DNA extraction kit Genomix (Biologica, Aichi, Japan)^7^. P/LP variants screening for *GJB2* and the mitochondrial DNA variants, m.1555A>G and m.3243A>G, were conducted for all subjects, as previously described^25^. For *EYA1* P/LP variants screening, the coding region (exons 2–16) was analyzed by Sanger sequencing using primers described in Supplementary Table S3. For subjects where no P/LP variants of *EYA1* was identified, P/LP variants screening of exons 1 and 2 of *SIX1* and exons 1–3 of *SIX5* was conducted by Sanger sequencing. For two patients (Family 10, 23), genomic DNA was subjected to targeted resequencing of 154 deafness genes using Nextseq 500 (Illumina, CA, USA), and the resulting data were analyzed as previously described^26^. The pathogenicity of each genetic variant was evaluated according to the guidelines by ClinGen Hearing Loss Clinical Domain Working Group^27^. For subjects where no P/LP variants of *EYA1* were identified by direct sequencing, MLPA was conducted to identify copy number variants of *EYA1*, using the SALSA MLPA KIT (P153; MRC-Holland, Amsterdam, the Netherlands). Results are presented using the International System for Human Cytogenomic Nomenclature (2016)^28^. Genetic analyses conducted for each subject are detailed in Supplementary Table S4.

### Statistics

Fisher’s exact test was used to evaluate contingency analyses. A p-value < 0.05 was considered significant. All statistical analyses were performed using Prism (GraphPad Software, Inc., CA, USA).

## Supplementary Information

Supplementary Information.

## Data Availability

Variant data is deposited to MGeND (Medical Genomics Japan Variant Database (https://mgend.med.kyoto-u.ac.jp/), applied for registration on November 10, 2021). The datasets generated and/or analyzed during the current study are available from the corresponding authors.
