# Supplementary material for: Phenotype–genotype correlation in patients with typical and atypical branchio-oto-renal syndrome
Source: Sci Rep. 2022 Jan 19;12:969. doi: 10.1038/s41598-022-04885-w (PMC8770796; doi:10.1038/s41598-022-04885-w)
Supplement: Supplementary file 1 — Supplementary Information. [file 41598_2022_4885_MOESM1_ESM.pdf]

Supplementary information

Phenotype–genotype correlation in patients with typical and atypical branchio-oto-renal syndrome

Masatsugu Masuda[1,2†], Ayako Kanno[2,3†], Kiyomitsu Nara[2], Hideki Mutai[2], Naoya Morisada[4], Kazumoto Iijima[5,6,7], Noriko Morimoto[8], Atsuko Nakano[9], Tomoko Sugiuchi[10], Yasuhide Okamoto[11], Sawako Masuda[12], Sayaka Katsunuma[13], Kaoru Ogawa[14], Tatsuo Matsunaga[2,15*]

***Corresponding author**: Tatsuo Matsunaga

Division of Hearing and Balance Research, National Institute of Sensory Organs, National Hospital Organization Tokyo Medical Center, 2-5-1 Higashigaoka Meguro-ku, Tokyo 152-8902, Japan

Tel: +81-3-3411-0111.　FAX: +81-3-3412-9811

Email: tatsuo.matsunaga@kankakuki.jp

† These authors contributed equally to this work.

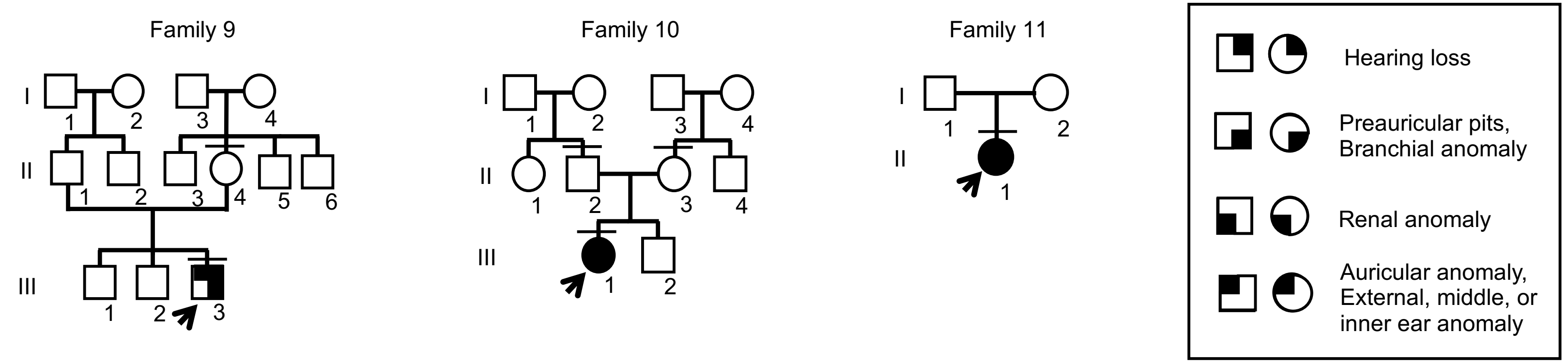

Supplementary figure S1. Pedigree of families with typical patients and without P/LP variants.

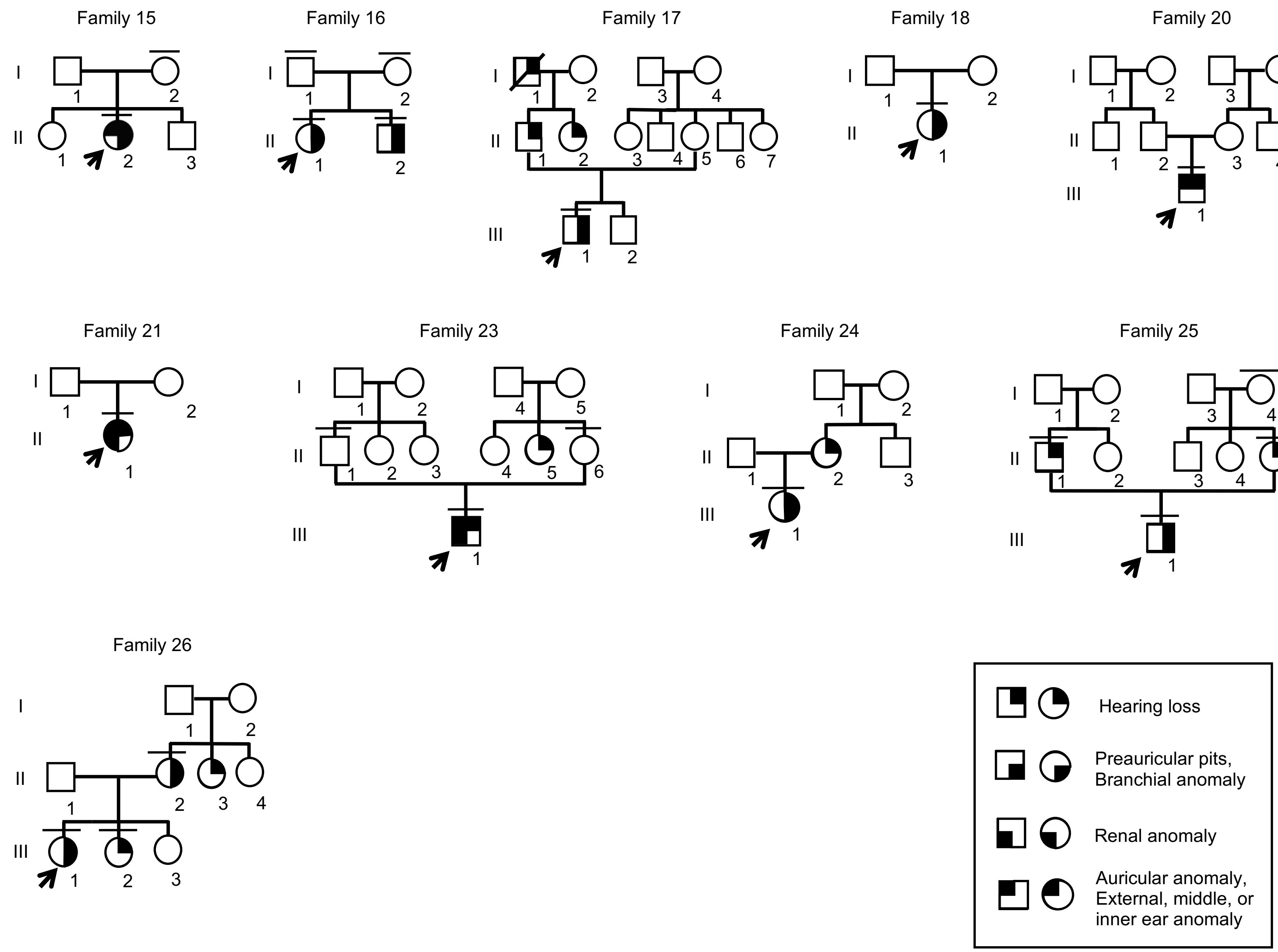

Supplementary figure S2. Pedigree of families with atypical patients and without P/LP variants.

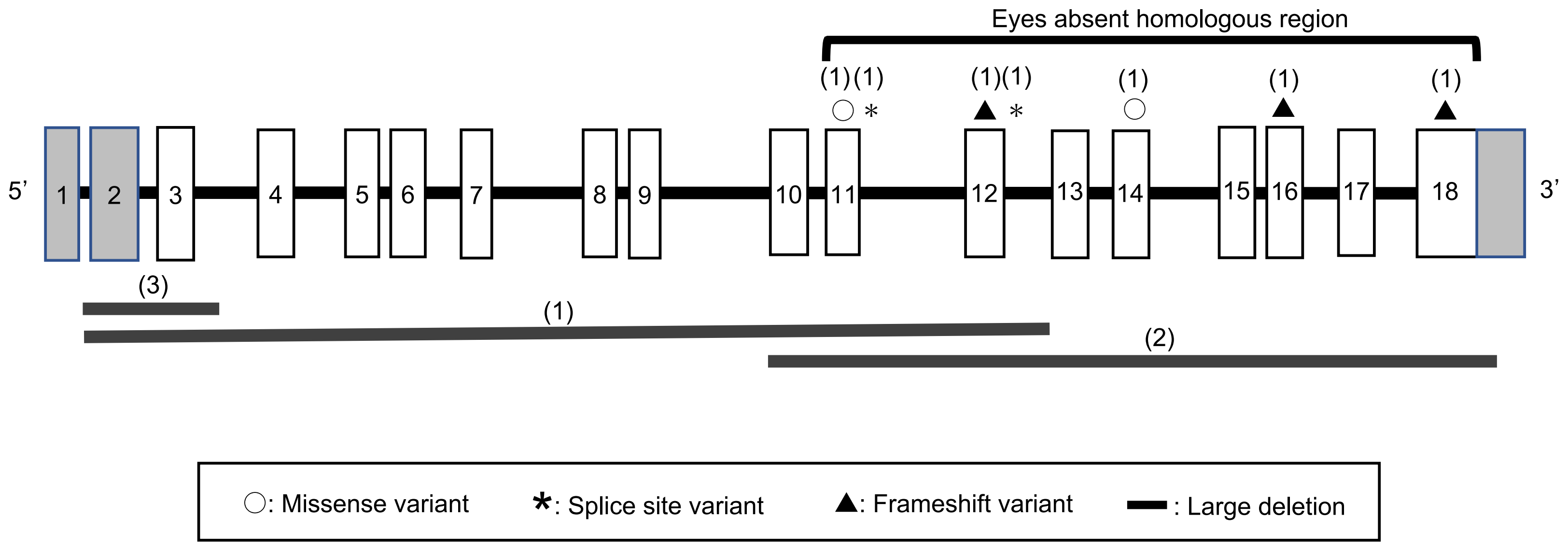

Supplementary figure S3. Location of *EYA1* P/LP variants detected in this study and the numbers of families with each variant. Exons are shown as rectangles numbered from 1 to 18. Numbers of families are shown in parentheses.

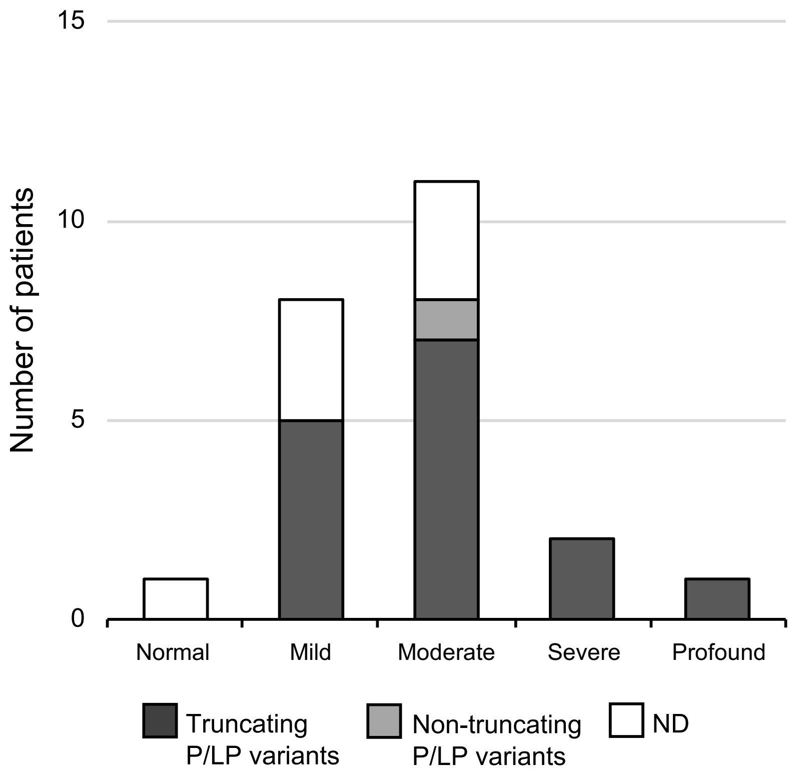

Supplementary figure S4. Association of hearing loss severity with P/LP variant type.

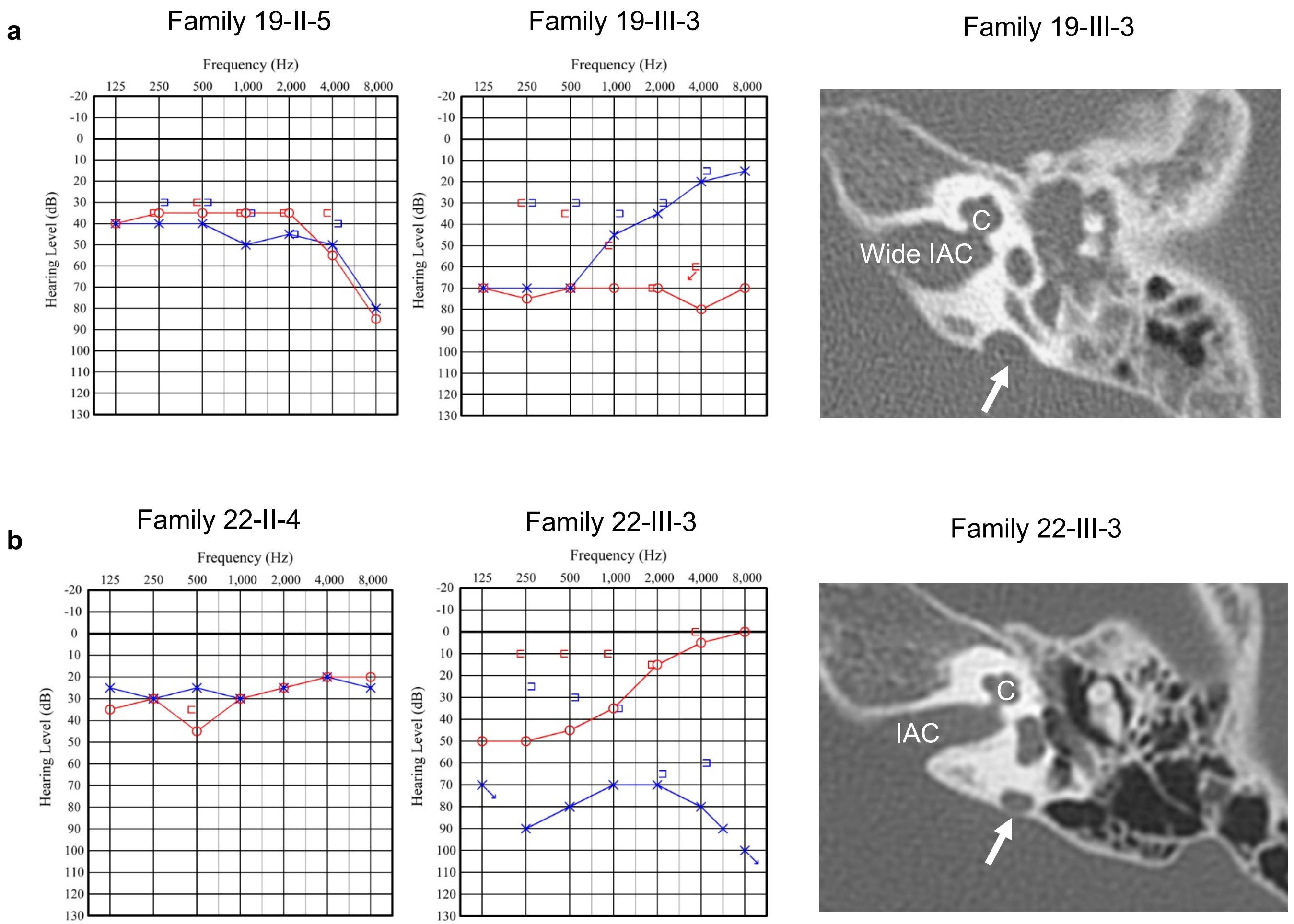

Supplementary figure S5. Case presentation of patients with atypical BOR syndrome with inner ear anomalies and a LP variant.
(a) and (b) Audiograms and temporal bone CT images of atypical patients with LP variants. C, hypoplastic cochlea; IAC, internal auditory canal; arrow, EVA.

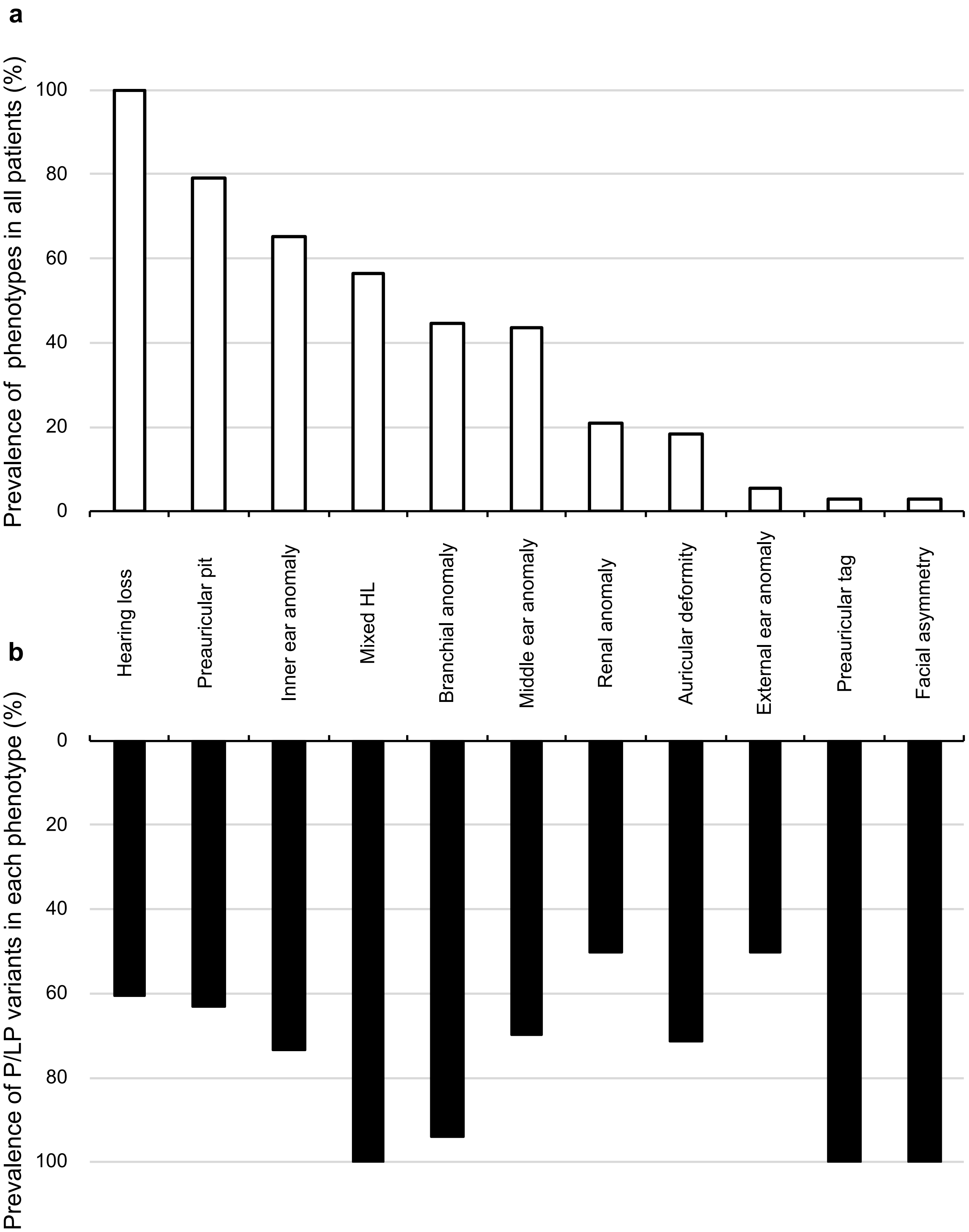

Supplementary figure S6. Prevalence of phenotypes in all patients (a) and detection rate of P/PL variants in each phenotype (b).

n = 38 except for mixed hearing loss, middle ear anomaly, and inner ear anomaly. n = 23 each for mixed hearing loss, middle ear anomaly, and inner ear anomaly.

Supplementary Table S1. Curation of variants

HGNC Gene Symbol	Variation	g.DNA (GRCh37/hg19)	cDNA/protein effect	gnomAD*	In silico analysis conservation (verPhCons)**	In silico analysis deleteriousness (CADD_PHRED)**	ACMG classification	Applied Evidence Codes
EYA1	NM_000503.5:c.1050+3G>T	NC_000008.10:g.72181972C>A	Splice site	Not reported	1	23.1	Likely Pathogenic	PS2_Moderate, PM2, PP3, PP4
EYA1	NM_000503.5:c.1141-1G>A	NC_000008.10:g.72129259C>T	Splice site	Not reported	1	35	Pathogenic	PVS1, PM2, PP4
EYA1	NM_000503.5:c.1766dup (p.Glu590Glyfs*42)	NC_000008.10:g.72111588dup	Frameshift	Not reported	1	33	Likely Pathogenic	PVS1_Strong, PM2, PP4
EYA1	NM_000503.5:c.1054_1055insG (p.Pro352Argfs*26)	NC_000008.10:g.72156923_72156924insC	Frameshift	Not reported	1	34	Pathogenic	PVS1, PM2, PP4
EYA1	NM_000503.5:c.979T>G (p.Trp327Gly)	NC_000008.10:g.72182046A>C	Missense	Not reported	1	31	Likely Pathogenic	PM1, PM2, PP3, PP4
EYA1	NM_000503.5:c.1487_1488del (p.Val496Glufs*35)	NC_000008.10:g.72127731_72127732del	Frameshift	Not reported	1	35	Pathogenic	PVS1, PM2, PP1_Supporting, PP4
EYA1	NM_000503.5:c.1319G>A (p.Arg440Gln)	NC_000008.10:g.72128968C>T	Missense	Not reported	1	33	Likely Pathogenic	PS2_Moderate, PS3_Supporting, PS4_Moderate, PM1, PM2, PP3, PP4
EYA1	rsa 8q13.3(EYA1exon10-18)x1	NC_000008.10:g.(?_72111499)_ (72183984_72211365)del	Deletion	Not reported	Not applied	Not applied	Likely Pathogenic	PVS1_Strong, PM2, PS4_Supporting, PP1_Supporting, PP4
EYA1	rsa 8q13.3(EYA1exon2-3)x1	NC_000008.10:g.(72246347_72267080)_ (72268698_?)del	Deletion	Not reported	Not applied	Not applied	Likely Pathogenic	PM2, PS4_Supporting, PP1_Moderate, PP4
EYA1	rsa 8q13.3(EYA1exon2-12)x1	NC_000008.10:g.(72129166_72156864)_ (72268698_?)del	Deletion	Not reported	Not applied	Not applied	Pathogenic	PVS1_Strong, PM2, PP4

*For short variants, both gnomAD v2.1.1 and v3.1.2 were searched[1]. For structural variants, gnomAD SVs v2.1 and the Database of Genomic Variants (DGV[2]) were searched. **CADD analysis was carried out in https://cadd.gs.washington.edu/score[3]. References: 1. Karczewski, K. J., et al. The mutational constraint spectrum quantified from variation in 141,456 humans. Nature 581, 434-443 (2020). 2. MacDonald, J. R., et al. The database of genomic variants: a curated collection of structural variation in the human genome. Nucleic Acids Res 42, D986-992 (2013). 3. Rentzsch, P., Schubach, M., Shendure, J., Kircher, M. CADD-Splice—improving genome-wide variant effect prediction using deep learning-derived splice scores. Genome Med 13, 31 (2021).

Supplementary Table S2. Inner, middle, and external ear anomalies of patients

Family no.	Patient no.	Findings
Typical BOR		
2	III-3	cochlear hypoplasia, ossicular malformation
3	III-1	cochlear hypoplasia, ossicular malformation
5	III-2	cochlear hypoplasia, enlarged vestibular aqueduct
6	II-2	cochlear hypoplasia, enlarged vestibule, ossicular malformation
	III-2	external auditory canal stenosis
7	II-1	cochlear hypoplasia
8	III-1	no anomaly
9	III-3	ossicular malformation
10	III-1	cochlear hypoplasia, ossicular malformation, external auditory canal stenosis
11	II-1	cochlear hypoplasia, enlarged vestibular aqueduct, enlarged vestibule
12	III-2	cochlear hypoplasia, enlarged vestibular aqueduct, ossicular malformation
13	III-2	cochlear hypoplasia, enlarged vascular aqueduct, enlarged vestibule, dysplastic semicircular canal, ossicular malformation
	III-1	cochlear hypoplasia, ossicular malformation
14	III-1	cochlear hypoplasia, dysplastic semicircular canal, ossicular malformation
Atypical BOR		
15	II-2	cochlear hypoplasia, enlarged vestibule, dysplastic semicircular canal
16	II-1	no anomaly
	II-2	no anomaly
18	II-1	no anomaly
19	III-3	cochlear hypoplasia, enlarged vestibular aqueduct, wide internal meatus
20	III-1	ossicular malformation
21	II-1	no anomaly
22	III-3	cochlear hypoplasia, enlarged vestibular aqueduct, dysplastic semicircular canal
23	III-1	common cavity, dysplastic semicircular canal

Supplementary Table S3. Primers for PCR amplification of *EYA1*, *SIX1*, and *SIX5*

EYA1

Exon	Primer	Sequence	product size	Sequence primer
2	EYA1ex2F	caggaaacagctatgaccGGCTGCTTTGCATGTTGATTGG	588	M13P5
	EYA1ex2R	tgtaaaacgacggccagtTCAAACCGAGGCTCCTATTCCC		
3	EYA1ex3F	caggaaacagctatgaccGTGCATATGATGAAGGGATACCTGAA	555	M13P5
	EYA1ex3R	tgtaaaacgacggccagtTGAAACGAAACCAATCTACGCAATG		
4	EYA1ex4aF	tgtaaaacgacggccagtGCCCTTGAGCATCAGGAAGACC	952	M13P1
	EYA1ex4aR	caggaaacagctatgaccTGGCGAGTCTGCTTAGAAGCGT		
5	EYA1ex5F	caggaaacagctatgaccCATGCCAGCAGGTATTTGCGA	582	M13P5
	EYA1ex5R	tgtaaaacgacggccagtTCACACAGAATTGACAAAGCACTGAA		
6	EYA1ex6F	caggaaacagctatgaccTCAGTGCTTTGTCAATTCTGTGTGAA	605	M13P5
	EYA1ex6R	tgtaaaacgacggccagtGCCCAGTGCTCAAATGGCAA		
7	EYA1ex7F	caggaaacagctatgaccGGGAAGCATTTGTAGTTAAACGGTTG	404	M13P5
	EYA1ex7R	tgtaaaacgacggccagtGGTGTCCTGCCTCTAAGCCCA		
8	EYA1ex8aF	tgtaaaacgacggccagtCAGGTGCTGCCATTCATTCTTC	891	M13P1
	EYA1ex8aR	caggaaacagctatgaccTAACTAAAGAGCTCAGACTTGC		
9	EYA1ex9F	caggaaacagctatgaccTGACAGACAATTCTTGTGTGGTTTGA	408	M13P1
	EYA1ex9R	tgtaaaacgacggccagtGAATGCCACAGTCATGCTGCTT		
10	EYA1ex10F	caggaaacagctatgaccGACGATGTAGGATTTCAGCAGAATGA	545	M13P5
	EYA1ex10R	tgtaaaacgacggccagtTTTAGAGAGAAACAGACCCTTTGCCA		
11	EYA1ex11bF	caggaaacagctatgaccCAGCCAAGGCAAAGACACATTGA	636	M13P1
	EYA1ex11bR	tgtaaaacgacggccagtCATTTAACCCAAGTAGCAAAGGGACA		
12	EYA1ex12F	caggaaacagctatgaccGCAGCTGTTGCCCAAGTCTCTC	587	M13P5
	EYA1ex12R	tgtaaaacgacggccagtTGCAAGTGTTACGAATTATGTTGGACA		
13,14	EYA1ex1314F	caggaaacagctatgaccTGCCACCTACTGATTGACATAGTTGAA	634	M13P5
	EYA1ex1314R	tgtaaaacgacggccagtGCCAGTGAGATGAAACTGCCCA		
15	EYA1ex15F	caggaaacagctatgaccAAATCTGGAGGCTGGTATTC	314	M13P5
	EYA1ex15R	tgtaaaacgacggccagtATGAACAAGCACGAGCATTGC		
16	EYA1ex16F	tgtaaaacgacggccagtGGCTGCAGTTGAGGGCCGAAA	582	M13P1
	EYA1ex16R	caggaaacagctatgaccATGCCCTGGTCTTAACGTTAGCCA		
17	EYA1ex17F	caggaaacagctatgaccTGCTGTGGCACATACAACCCA	517	M13P5
	EYA1ex17R	tgtaaaacgacggccagtGAACACCACTTGTGGCCCTTGA		
18	EYA1ex18F	caggaaacagctatgaccCACTGGATACGTGAACAGCTGCC	574	M13P5
	EYA1ex18R	tgtaaaacgacggccagtCAGCAACTGCGCATCACCAG		

#Sequeces of the sequence primers are as follows: M13P5, CAGGAAACAGCTATGAC; and M13P1:GTAAAACGACGGCCAGT.
Nucleotides in lower case indicate sequencing primers.

SIX1

Exon	Primer	Sequence	product size	Sequence primer
1a	SIX1-F1	AGGAGGAGGTTAGGAACAGC	499	SIX1-F1
	SIX1-R1	GTAGCTGGTCTCCTCGCCGTC		
1b	SIX1-ex1b-F	CTGGAGAGCCACCAGTTCTC	414	SIX1-ex1b-R
	SIX1-ex1b-R	GGGCGGAGGAGAAAGGAC		
2a	SIX1-ex2a-F	CCCTCCCTCCATCTCACAC	346	SIX1-ex2f-1, SIX1-ex2a-R
	SIX1-ex2a-R	CCGAGCAGAGAGTCTTGGAG		
2b	SIX1-ex2b-F	TCTCTCCCGGGCTTAACAG	457	SIX1-ex2b-F, SIX1-ex2r-1
	SIX1-ex2b-R	AGAAAGGCTGCTGAAACAGG		

#Sequeces of the sequence primers are as follows: SIX1-ex2f-1, AACCATATGGTGTTGTCCTC; and SIX1-ex2r-1, AGTGTCCCTAGTCGCTG.

SIX5

Exon	Primer	Sequence	product size	Sequence primer
1	SIX5-ex1-f4	GAGTGGAGAGCTGGATCTCG	1109	SIX5-ex1-f4, SIX5-ex1-rb7
	SIX5-ex1-rb7	GCCTGAGATTGTGAGCTGGT		
2	SIX5-ex2-f4	GAAACTTGGGCCAGGGAAAG	973	SIX5-ex2-f4, SIX5-ex2-r1
	SIX5-ex2-r1	CTCCCTCTCCGAGATGACTG		
3	SIX5-ex3-f4	AGCTGCACAGTCTCCACTTC	797	SIX5-ex3-f4, SIX5-ex3-r4
	SIX5-ex3-r4	GTGGTGACTGGGGTCTTCAG		

Supplementary Table S4. Genetic analysis methods conducted for each patient

Family no.	Patient no.	Symptom of BOR syndrome	*EYA 1* Sanger sequencing / NGS	*EYA 1* MLPA	*SIX1* Sanger sequencing / NGS	*SIX5* Sanger sequencing / NGS
1	III-3	+	+	+		
	II-3	-	+			
	II-1	-	+			
2	IV-1	+	+	+	+	+
	II-3	+	+	+	+	+
3	III-1	+	+			
4	IV-2	+	+			
	III-2	+	+			
	IV-1	-	+			
5	III-2	+	+			
	II-5	+	+			
6	II-2	+	+	+	+	+
	III-2	+		+		
	I-2	+		+		
7	II-1	+	+	+		
8	III-1	+	+	+		
	II-2	+		+		
	II-3	-		+		
9	III-3	+	+	+	+	+
10	III-1	+	+	+	+	+
11	II-1	+	+	+	+	+
12	III-2	+	+			
13	III-2	+	+			
	III-1	+	+			
	II-3	+	+			
	II-2	-	+			
14	III-1	+	+			
	II-2	-	+			
	II-3	-	+			
15	II-2	+	+	+	+	+
16	II-1	+	+	+	+	+
	II-2	+	+	+	+	+
17	III-1	+	+	+	+	+
18	II-1	+	+	+	+	+
19	III-3	+	+	+	+	+
	II-5	+		+		
	II-2	-		+		
20	III-1	+	+	+	+	+
21	II-1	+	+	+	+	+
22	III-3	+	+	+	+	+
	II-4	+		+		
	II-5	-		+		
23	III-1	+	+	+	+	+
24	III-1	+	+	+	+	+
25	III-1	+	+	+	+	+
26	II-2	+	+	+	+	+
	III-1	+	+	+	+	+
